# Der Brexit-Prozess und die Austrittsdoktrin: Die Führungsrolle des Europäischen Rats

**DOI:** 10.1007/s12399-021-00842-z

**Published:** 2021-04-01

**Authors:** Birgit Bujard, Wolfgang Wessels

**Affiliations:** 1 Köln, Deutschland; 2 CETEUS, Universität zu Köln, Gottfried-Keller-Str. 1, 50931 Köln, Deutschland

**Keywords:** Europäischer Rat, Brexit, EU-Austritt, Austrittsdoktrin, EU-britische Beziehungen, European Council, Brexit, Withdrawal, Exit doctrine, EU-British relations

## Abstract

Zum Verständnis des Brexit-Prozesses ist eine Analyse der Rolle des Europäischen Rats unumgänglich. Er hat bei der Gestaltung der Beziehung zum Vereinigten Königreich die Führung übernommen und zentrale Entscheidungen zum Verfahren und den Unionszielen getroffen. Er hat sie in ein Narrativ gefasst, das die Errungenschaften der EU und die Unterschiede zwischen Mitgliedern und Nichtmitgliedern nachhaltig hervorhebt. Wie die Beitrittskriterien könnten diese Vorgaben und Beschlüsse zur Austrittsdoktrin werden.

## Abstract (en)

To understand the Brexit process an analysis of the European Council’s role is essential. In the process of shaping the relationship with the United Kingdom it assumed the lead and took key decisions on the negotiating procedure and the EU’s goals. It framed its decisions in a narrative, which highlighted the EU’s achievements and the differences between members and non-members. Similar to the membership conditions these provisions and decisions could become the EU’s exit doctrine.

## Der Europäische Rat und der schwierige Partner

Im Verhältnis zur Europäischen Gemeinschaft (EG) und späteren Europäischen Union (EU) galt das Vereinigte Königreich schon früh als „schwieriger Partner“ (George 1998, S. 1, eigene Übersetzung). Zwar hatte Winston Churchill 1946 die Schaffung der „Vereinigten Staaten von Europa“ gefordert, sah sein Land aber nicht als Teil davon (Churchill zit. n. Gastgeyer 2005, S. 47, 49). Zu Beginn des Einigungsprozesses entschied sich das Land dann auch gegen eine Beteiligung. Auch nach dem Beitritt 1973 zeigte es sich der weiteren Integration häufig zögerlich bis ablehnend gegenüber und präferierte ein Verhältnis, wie es Margaret Thatcher 1988 definierte: die „willige und aktive Kooperation zwischen unabhängigen souveränen Staaten ist der beste Weg, um eine erfolgreiche Europäische Gemeinschaft aufzubauen“ (Thatcher 1988, eigene Übersetzung).

Der Europäische Rat beziehungsweise die Staats- und Regierungschefs^1^ der Mitgliedstaaten haben seit den 1960er-Jahren eine zentrale Rolle bei der Bestimmung des Verhältnisses zwischen EG/EU und dem Vereinigten Königreich gespielt. So haben die Staats- und Regierungschefs der sechs Gründerstaaten 1969 auf dem Gipfel in Den Haag, den man als Vorläufer des Europäischen Rates sehen kann, die Aufnahme des Landes beschlossen, nachdem dessen erste beiden Beitrittsgesuche am Widerstand Charles de Gaulles gescheitert waren (Knipping 2004, S. 156–157). Seit seiner Gründung 1974 hat der Europäische Rat immer wieder Beschlüsse zu besonderen Anliegen des Königreichs getroffen: Neben der regelmäßigen Beschäftigung im Rahmen der Haushaltsbeschlüsse vereinbarten seine Mitglieder gemeinsam auch Ausnahmeregeln für das Land – etwa zur Wirtschafts- und Währungsunion Anfang der 1990er-Jahre und später zum Schengenraum (Wessels 2016, Kap. 4). Diese Opt-outs waren Ausdruck einer differenzierten Integration (Tekin 2020, S. 2), die die anderen Staaten zuließen, um sich selbst weiter integrieren zu können.

Der Europäische Rat fasste auch die Beschlüsse zu den Forderungen von Premierminister David Cameron nach Vertragsänderung, nachdem dieser seinen Wählern 2013 ein Referendum über die Mitgliedschaft infolge einer Neuverhandlung des Verhältnisses zur EU versprochen hatte. In intensiven Verhandlungen beschloss das Gremium im Februar 2016 Formulierungen, die Cameron die Kampagne für den EU-Verbleib erleichtern sollten. Die 27 anderen Staats- und Regierungschefs boten jedoch nur weitgehend kosmetische Anpassungen bei Verfahren an und keine Vertragsänderung bezüglich der Grundpfeiler der Union (Europäischer Rat 2016a).

Um die Verhandlungen mit dem Vereinigten Königreich nach dem Referendum vom 23. Juni 2016, bei dem 51,9 % der britischen Bürger für den Austritt stimmten, auf EU-Seite zu verstehen, ist eine Analyse des Europäischen Rats unumgänglich.^2^ Anhand dessen Schlussfolgerungen untersucht dieser Beitrag seine Rolle in den Verhandlungen. Er hat, wie im Folgenden gezeigt wird, die Führung bei der Gestaltung der künftigen Beziehung zum Königreich eingenommen und prägende Entscheidungen zum Verfahren und zu den Unionszielen für diese Beziehung getroffen. Letztere spiegeln sich auch im Handels- und Kooperationsabkommen von Dezember 2020 wider. Wie die Analyse der Schlussfolgerungen von 2016 bis 2020 zeigt, hat er seine Festlegungen in ein Narrativ gefasst, dass die Errungenschaften der Union sowie die Unterschiede zwischen Mitgliedern und Nichtmitgliedern nachhaltig hervorhob. Analog zu den Beitrittskriterien, die er 1993 formulierte, könnten diese Vorgaben und Beschlüsse zur „Austrittsdoktrin“ (Lippert 2019, S. 632; von Ondarza 2020, S. 92) werden.

## Der Europäische Rat im Brexit-Prozess: Ein Rückblick auf seine Schlussfolgerungen

Zwischen Ende Juni 2016 und dem EU-Austritt des Vereinigten Königreichs am 31. Januar 2020 befasste sich der Europäische Rat bei 19 von 38 seiner Sitzungen mit dem britischen Austritt und dem künftigen Verhältnis zum Land. Die Beratungen des Europäischen Rats zu Austrittsverfahren und Verhandlungsposition der Union fanden ohne britische Beteiligung statt (Tab. 1).

**Table 1:** 

23.6.2016	Referendum im Vereinigten Königreich (VK): 51,9 % der Bürger stimmen für EU-Austritt
28./29.6.2016	Europäischer Rat diskutiert Konsequenzen des Referendumsergebnisses
15.12.2016	Europäischer Rat legt Verfahren für Verhandlungen mit VK fest
29.3.2017	VK stellt EU-Austrittsgesuch gemäß Art. 50 EUV
29.4.2017	Europäischer Rat verabschiedet Leitlinien für die Austrittsverhandlungen
19.6.2017	Beginn der Austrittsverhandlungen zwischen EU und VK
15.12.2017	Europäischer Rat beschließt ausreichende Fortschritte in erster Verhandlungsphase und Beginn der zweiten Phase
23.3.2018	Europäischer Rat verabschiedet Leitlinien für die Verhandlungen zu künftigen Beziehungen
19./20.9.2018	Europäischer Rat bestätigt, dass es kein Austrittsabkommen ohne Lösung für Irland geben wird, beschließt Abschluss einer Politischen Erklärung über künftige Beziehung mit VK
14.11.2018	EU und VK (unter Premierministerin Theresa May) einigen sich auf Austrittsabkommen & Politische Erklärung zu künftigen Beziehungen
25.11.2018	Europäischer Rat billigt Austrittsabkommen & Politische Erklärung
13.12.2018	Europäischer Rat bestätigt Austrittsabkommen & Politische Erklärung
22.3.2019	Europäischer Rat beschließt erste Fristverlängerung der Artikel 50-Verhandlungen
10.4.2019	Europäischer Rat beschließt zweite Fristverlängerung der Artikel 50-Verhandlungen
Sommer – Herbst 2019	Nachverhandlungen zwischen EU und VK (unter Premierminister Boris Johnson) über Nordirland-Protokoll des Austrittsvertrags
17.10.2019	EU und VK einigen sich auf geändertes Austrittsabkommen & Politische Erklärung, Europäischer Rat billigt Abkommen & Politische Erklärung
29.10.2019	Europäischer Rat beschließt dritte Fristverlängerung der Artikel 50-Verhandlungen
13.12.2019	Europäischer Rat beginnt Verfahrensschritte für Verhandlungen über künftige Beziehungen
30.1.2020	Ratifikation des Austrittsabkommens ist abgeschlossen
1.2.2020	EU-Austritt des VK, Beginn der Übergangsphase
25.2.2020	Rat der Europäischen Union verabschiedet Verhandlungsmandat für künftige Beziehungen
2.3.2020	Beginn der Verhandlungen über EU-VK Partnerschaftsabkommen
15./16.10.2020	Europäischer Rat sieht Fortschritte in den Verhandlungen als nicht ausreichend für Einigung
24.12.2020	EU und VK einigen sich auf Handels- und Kooperationsabkommen
1.1.2021	Ende der Übergangsphase, Abkommen tritt vorläufig in Kraft

### Die Selbstermächtigung des Europäischen Rats zum Herrn des Verfahrens

Der Vertrag über die Europäische Union (EUV) weist dem Europäischen Rat eine konkrete Rolle im Austrittsprozess zu: Ein austrittswilliger Staat muss seine Austrittsnotiz an den Europäischen Rat senden. Dieser erlässt Leitlinien für die Austrittsverhandlungen. Über eine mögliche Verlängerung der zweijährigen Verhandlungsfrist entscheidet er auch einstimmig (Art. 50 EUV).

Der Europäische Rat traf sich unmittelbar nach dem Referendum am 28./29. Juni 2016 und diskutierte erstmals zu 27 ohne den britischen Premier mögliche Konsequenzen des Ergebnisses für die Union. Mit dieser Sitzung nahm er die Führung beim folgenden Brexit-Prozess ein, indem er zentrale Aspekte der Unionsreaktion festlegte und sich selbst eine wichtige Rolle im Verfahren gab. Er bekräftigte, dass der britische Austritt in „geordneter Weise“ auf Basis von Art. 50 EUV stattfinden müsse (Europäischer Rat 2016b, S. 1). Solange das Königreich Mitglied sei, würden sich dessen Verpflichtungen und Rechte nicht ändern. Das Land solle möglichst bald den Austrittsprozess mit der Stellung einer Austrittsnotiz einleiten. Ohne dies könne „es keine wie auch immer gearteten Verhandlungen geben“ (Europäischer Rat 2016b, S. 1). Bei Vorliegen der Notiz werde der Europäische Rat die Leitlinien für einen Austrittsvertrag festlegen. Der Europäischen Kommission und dem Europäischen Parlament wies er zu, „ihre Rolle im Einklang mit den Verträgen in vollem Umfang wahr[zu]nehmen“ (Europäischer Rat 2016b, S. 1).

Am 15. Dezember 2016 traf der Europäische Rat weitere Verfahrensbeschlüsse und bestätigte seine Schlüsselrolle bei der Setzung der EU-Position. Er kündigte die regelmäßige Befassung mit den Austrittsverhandlungen an. Nach seinem Beschluss der Leitlinien werde der Rat den Verhandlungsstart auf Empfehlung der Europäischen Kommission beschließen und Richtlinien für die Verhandlungen auf Basis dieser Leitlinien erlassen. Der Europäische Rat rief den Rat der Europäischen Union (Rat) auf, der Kommission die Verhandlungsführung zu übertragen. Er erklärte zudem, Vertreter des Präsidenten des Europäischen Rats würden an allen Verhandlungssitzungen teilnehmen (Europäischer Rat 2016d, S. 2). Auch regelte er die enge Einbindung des Europäischen Parlaments, dessen Zustimmung nach Art. 50 EUV zum Austrittsvertrag nötig ist, in das Verfahren: Es sollte kontinuierlich durch den Verhandlungsführer über die Verhandlungen informiert werden. Zudem lud er den Parlamentspräsidenten zu seinen Sitzungen ein (Europäischer Rat 2016d, S. 3).

Infolge der im März 2017 gestellten britischen Austrittsnotiz verabschiedete der Europäische Rat am 29. April 2017 seine Leitlinien und spezifizierte das Verfahren: Die Verhandlungen würden zweiphasig sein. Erst würden die wichtigsten Themen des Austritts geklärt. Anschließend würden Gespräche über die künftige Beziehung begonnen, deren formeller Abschluss aber erst nach dem britischen EU-Austritt möglich sei. Auch hier gab sich das Gremium eine Führungsrolle, da es sich die Entscheidung übertrug, festzulegen, wann die Ergebnisse der ersten Phase hinreichend seien, um die zweite zu beginnen (Europäischer Rat 2017b, S. 4). Die folgenden Verhandlungen waren schwierig. Doch im Dezember 2017 beschloss der Europäische Rat, der Verhandlungsstand sei hinreichend, um mit der zweiten Phase zu starten (Europäischer Rat 2017c, S. 1). Das größte Problem der Austrittsverhandlungen war die Frage, wie man nach einem britischen Austritt aus EU-Zollunion und Binnenmarkt die Grenze zwischen Nordirland und der Republik Irland gemäß des 1998er Friedensabkommens offenhalten könnte. Nach weiteren Verhandlungen – die den Abschluss eines Abkommens, das im Unterhaus auch wegen der Regelung zur Lösung dieser Frage dreimal scheiterte, einen Premierministerwechsel und mehrere Verlängerungen der zweijährigen Artikel-50-Frist beinhalteten – einigte man sich im Herbst 2019 auf einen geänderten Vertrag (Bujard 2019). Diesem zufolge würde Nordirland – anders als der Rest des Landes – auch nach dem Austritt EU-Binnenmarktregeln im Güterbereich und EU-Zölle anwenden. So würde die Grenze auf der irischen Insel offenbleiben können, aber eine Zoll- und regulatorische Grenze zwischen Großbritannien und Nordirland entstehen (Bujard 2020, S. 430). Die Lösung der Grenzfrage war deshalb wichtig, weil sie das Mitglied Irland betraf, das eine Landesgrenze mit dem Königreich teilte und am stärksten die ökonomischen Auswirkungen des britischen Austritts zu spüren bekommen würde (Europäisches Parlament 2017, S. 32). Am 17. Oktober 2019 nahm der Europäische Rat das Abkommen und die geänderte Politische Erklärung, eine Absichtserklärung zur künftigen Beziehung, an (Europäischer Rat 2019c, S. 1).

Am 13. Dezember 2019 bestätigte der Europäischen Rat seine Rolle als Herr des Verfahrens bei der Festlegung des Verhandlungsprozesses über die künftige Beziehung: Er forderte die Europäische Kommission auf, nach dem britischen Austritt zügig ein Verhandlungsmandat vorzulegen, und den Rat, dieses bald anzunehmen (Europäischer Rat 2019d, S. 1).

### Die nachdrückliche Vorgabe der EU-Verhandlungsposition

Neben der Strukturierung des Verfahrens nahm der Europäische Rat auch die Beschlussfassung der Verhandlungsposition vor und zeigte, dass er vertragsgemäß die „politischen Zielvorstellungen“ (Art. 15 (1) EUV) für die EU vorgeben würde. Beginnend mit der ersten Sitzung nach dem Referendum sowie im September 2016 in Bratislava fasste er seine Beschlüsse in ein Narrativ, das die Errungenschaften der EU und die Unterschiede zwischen Mitgliedern und Nichtmitgliedern hervorhob.

Bei der Einordnung der Auswirkungen des Referendums auf den Einigungsprozess bekräftigten die verbleibenden Mitglieder im Juni 2016, „vereint zu bleiben und im Rahmen der EU zusammenzuarbeiten, um die Herausforderungen des 21. Jahrhunderts zu bewältigen und Lösungen im Interesse unserer Nationen und Völker zu finden“ (Europäischer Rat 2016b, S. 2). In Bratislava machte er deutlich, dass er das britische Votum nicht als Grund für ein Ende der Integration sah: „Aufbauend auf diese gemeinsame Geschichte sind wir entschlossen, die EU mit 27 Mitgliedstaaten zum Erfolg zu führen“ (Europäischer Rat 2016c, S. 1).

In ihren Erklärungen von Juni und September 2016 legten die 27 den Fokus auf die Errungenschaften der Union und bekräftigen ihren Entschluss am EU-Projekt festzuhalten: „Die Europäische Union ist eine historische Leistung, die Frieden, Wohlstand und Sicherheit auf dem europäischen Kontinent gebracht hat, und wird unser gemeinsamer Rahmen bleiben“ (Europäischer Rat 2016b, S. 2; auch 2016c, S. 1).

Der Europäische Rat ging auch auf Defizite der EU ein: „Die europäischen Bürger erwarten von uns bessere Ergebnisse, wenn es darum geht, Sicherheit, Beschäftigung und Wachstum zu gewährleisten und Hoffnung auf eine bessere Zukunft zu geben“ (Europäischer Rat 2016b, S. 2). Er kündigte im Juni 2016 den Start einer „politischen Reflexion“ an, „um Impulse für weitere Reformen im Einklang mit unserer strategischen Agenda und für die Weiterentwicklung der EU der 27 Mitgliedstaaten zu geben“ (Europäischer Rat 2016b, S. 2). Mit diesen Erklärungen sowie denen von Rom 2017, Sibiu 2019 und der strategischen Agenda 2019–2024 (Europäischer Rat 2017a, 2019a, b) wiesen die 27 Staats- und Regierungschefs dem Brexit nur einen begrenzten Stellenwert für den weiteren Integrationsprozess zu.

Im April 2017 und März 2018 beschlossen sie die Leitlinien für die Verhandlungen über Austritt und künftige Beziehung. Bereits im Juni 2016 hatten sie erste Festlegungen gemacht: Eine differenzierte Integration mit Opt-ins als Nichtmitglied analog zu den Opt-outs während der Mitgliedschaft würde etwa beim Binnenmarkt nicht möglich sein: „Voraussetzung für den Zugang […] ist, dass alle vier Freiheiten akzeptiert werden“ (Europäischer Rat 2016b, S. 2). Zudem benötige ein Vertrag mit dem Königreich ein „ausgewogene[s] Verhältnis zwischen Rechten und Pflichten“ (Europäischer Rat 2016b, S. 2).

In seinen 2017er Leitlinien bekräftigte der Europäische Rat das Ziel einer engen Beziehung bei Handel, Terrorismus- und Kriminalitätsbekämpfung sowie Außen‑, Sicherheits- und Verteidigungspolitik (Europäischer Rat 2017b, S. 8; auch 2018a, S. 2). Jedoch, so erklärte er erneut, müsse „jedes Abkommen […] auf einem ausgewogenen Verhältnis zwischen Rechten und Pflichten beruhen [...], wobei faire Wettbewerbsbedingungen sicherzustellen sind“ (Europäischer Rat 2017b, S. 3; auch 2018a, S. 3). Zudem differenzierte er klar zwischen Mitgliedern und Drittstaaten: „Die Beziehungen zwischen der Union und einem Nicht-Mitgliedstaat können nicht dieselben Vorteile bieten wie eine Unionsmitgliedschaft“ (Europäischer Rat 2017b, S. 8; auch 2018a, S. 3).

Bezüglich der britischen Absicht, den Binnenmarkt zu verlassen, erklärte sich das Gremium bereit, mit dem Land nach dessen Austritt über ein Freihandelsabkommen zu verhandeln. Ein solches könne aber „nicht auf eine Beteiligung am Binnenmarkt oder an Teilen davon hinauslaufen, da dies dessen Integrität und reibungsloses Funktionieren untergraben würde“ (Europäischer Rat 2017b, S. 8). 2018 erklärte er erneut, „dass die vier Freiheiten unteilbar sind und es kein ‚Rosinenpicken‘ geben kann, d. h. eine Beteiligung am Binnenmarkt lediglich in einzelnen Sektoren, die die Integrität und das ordnungsgemäße Funktionieren des Binnenmarktes untergraben würde“ (Europäischer Rat 2018a, S. 3). Zudem verwiesen die Staats- und Regierungschefs auf die Beschlussfassungsautonomie der Union und die ebenfalls autonome Rolle des Gerichtshofs der Europäischen Union. Separate Einigungen über einzelne Politikfelder lehnten sie ebenso ab wie bilaterale Verhandlungen mit dem Königreich (Europäischer Rat 2017b, S. 3).

In seinen 2018er Leitlinien spezifizierte der Europäische Rat die Unionsposition zur künftigen Beziehung. Kernpunkte waren das Ziel eines Freihandelsabkommens für den Warenhandel ohne Zölle und Quoten, aber mit angemessenen Ursprungsregeln. Auch für den Dienstleistungshandel wolle man eine Einigung. Hier würde der Umfang allerdings begrenzter sein, da das Königreich ein Drittstaat sein würde. Bei der Fischerei sprach sich das Gremium für die Beibehaltung des aktuellen Zugangs zu gegenseitigen Gewässern und bestehender Quoten aus (Europäischer Rat 2018a, S. 4). Ferner wolle die EU eine justizielle Zusammenarbeit in Strafsachen und Strafverfolgung. Auch diese müsse berücksichtigen, dass das Land ein Drittstaat und kein Schengenland sei. Ebenso nannte der Europäische Rat erneut als Ziel die „enge Zusammenarbeit […] in der Außen‑, Sicherheits- und Verteidigungspolitik“ (Europäischer Rat 2018a, S. 6).

Nach dem britischen EU-Austritt am 31. Januar 2020 begannen im März die formellen Verhandlungen über das künftige Verhältnis. Zuvor hatte der Rat den von der Europäischen Kommission vorgelegten und auf den Leitlinien des Europäischen Rates basierenden Entwurf für ein Verhandlungsmandat angenommen (Rat der Europäischen Union 2020).

## Die Handschrift des Europäischen Rats im Handels- und Kooperationsabkommen

Das zwischen EU und Vereinigtem Königreich nach schwierigen Verhandlungen im Dezember 2020 geschlossene Abkommen trägt an vielen Stellen die Handschrift des Europäischen Rats. Es zeigt die hohen Schutzvorkehrungen, die er für den Binnenmarkt getroffen hat. Es ist das angebotene Freihandelsabkommen ohne Zölle und Quoten, aber mit Ursprungsregeln im Güterhandel. Ergänzt wird es durch *Level Playing Field*-Regelungen, die weiter gehen als andere EU-Handelsverträge. Es enthält zwar Vereinbarungen zum Dienstleistungshandel, die allerdings kaum über das hinausgehen, was neuere EU-Handelsabkommen üblicherweise umfassen (Melo Araujo 2021). Eine britische Binnenmarktbeteiligung ohne die Beibehaltung der vier Freiheiten ist also nicht möglich. Beide Seiten arbeiten weiter in Fragen der inneren Sicherheit zusammen, doch spiegelt sich im Abkommen, wie der Europäische Rat zuvor formuliert hatte, dass das Königreich ein Drittstaat und kein Schengenland ist (Institute for Government 2021). Noch offene Äquivalenzentscheidungen zu Finanzdienstleistungen und Datenschutz trifft die Union unverändert unilateral (Bille und Morin 2021).

Doch der Europäische Rat erreichte nicht alle seine Ziele. So regelt das Abkommen keine Fragen der Außen‑, Sicherheits- und Verteidigungspolitik. London hatte das abgelehnt (Bille und Morin 2021). Ferner gibt es keine Rolle für den Gerichtshof der Europäischen Union in der Governancestruktur und bei der Streitschlichtung, wie es beim Austrittsvertrag der Fall ist (Fella et al. 2020, S. 15). Auch bei der Fischerei erreichte die EU ihre Maximalposition einer Beibehaltung bestehender Regelungen nicht. Allerdings ist die Einigung näher an dem, was die EU wollte, als an dem, was das Vereinigte Königreich ursprünglich als Ziel formulierte (Stewart 2020).

## Überraschender Zusammenhalt

Angesichts üblicher Kontroversen bei zentralen Gestaltungfragen der Union ist es überraschend, dass die Beratungen im Europäischen Rat entgegen landläufiger Erwartungen beim Brexit nicht zu einem Auseinanderfallen der 27 sondern zu einem hohen Grad von Geschlossenheit führten. Die enge Koordinierung auf höchster politischer Ebene machte es möglich. Diese Geschlossenheit gab London auch keine Chance der Spaltung der Union im Verhandlungsverlauf und trug dazu bei, dass der Europäische Rat seine Schlüsselrolle so erfolgreich einnehmen konnte. Die 27 Staats- und Regierungschefs blieben bei der gemeinsam beschlossenen Ablehnung bilateraler Verhandlungen, vertraten die EU-Position und gingen nicht auf britische Versuche ein, an der Kommission vorbei zu verhandeln. So auch die großen Mitgliedstaaten: Bei Boris Johnsons Antrittsbesuchen im August 2019 betonten Angela Merkel und Emmanuel Macron hinsichtlich dessen Ruf nach Streichung der bestehenden Nordirland-Regelung die EU-Position und verhandelten nicht unilateral (Kahlweit 2019). Die EU-Mitglieder solidarisierten sich früh mit Irland und nur dessen Premier Leo Varadkar verhandelte im Herbst 2019 bilateral mit Johnson über die irische Grenzfrage, was den Abschluss eines geänderten Vertrags ermöglichte (Fleming et al. 2019).

Bei den Verhandlungen über das künftige Verhältnis wurde eine brüchigere Front erwartet, da die Länder unterschiedlich stark von neuen Regeln etwa bei Fischerei oder Handel betroffen sein würden. Doch bis zum Verhandlungsende hielten die Staats- und Regierungschefs an ihren Leitlinien fest.

Anders als bei den Verhandlungen über den Austritt befasste sich der Europäische Rat nicht so intensiv mit denjenigen über die künftige Beziehung – nur bei vier seiner 13 Sitzungen im Jahr 2020. Bei der umfangreichsten Diskussion im Oktober trafen die Staats- und Regierungschefs besondere Vorkehrungen, um diese vertraulich zu halten: Die Beteiligten durften währenddessen keine Mobilgeräte dabeihaben (Eder 2020). Dass sie 2020 weniger Zeit für Debatten über das Verhältnis zum Königreich aufbrachten, hatte mehrere Gründe. Zum einen gab es Dringlicheres: An erster Stelle stand der Umgang mit den gesundheitspolitischen und ökonomischen Auswirkungen der COVID-19-Pandemie. Zudem war die Verabschiedung des Mehrjährigen Finanzrahmens für 2021–2027 und eines europäischen Corona-Wiederaufbaufonds schwierig und langwierig. Die erst im Dezember 2020 erfolgte Einigung darüber war zuvor durch Ungarn und Polen wegen des neuen Rechtsstaatlichkeitsmechanismus zum Schutz des EU-Haushalts blockiert worden (Gnauck et al. 2020). Allerdings scheinen die 27 auch keine Notwendigkeit der Änderung ihrer Leitlinien gesehen zu haben.

Im Herbst 2020 zeigten sich zwar Differenzen. Diese führten aber nicht zur Spaltung und Verhandlungsalleingängen. So forderten die Staats- und Regierungschefs Frankreichs, Belgiens und der Niederlande im November die Publikation von Notfallmaßnahmen im Fall eines *No-Deals*. Die Europäische Kommission hatte dies bis dahin nicht getan, um zu vermeiden, dass London es als Indiz sehen könnte, die EU habe die Hoffnung auf eine Einigung aufgegeben (Moens und Von der Burchard 2020). Auch bei den noch offenen Themen wurden Unterschiede sichtbar. So erklärte etwa Frankreich im Oktober, es sei nicht bereit auf Kosten der eigenen Fischer ein Abkommen zu schließen (Moens 2020). Doch auch hier kam es nicht zum Bruch. Auch nach dem ersten mehrerer Gespräche zwischen Kommissionspräsidentin Ursula von der Leyen und Johnson im Dezember forderte der britische Premier erneut bilaterale Verhandlungen mit Macron und Merkel, was von Unionsseite abgelehnt wurde (Boffey und Elgot 2020).

## Fazit: Auf dem Weg zu einer Austrittsdoktrin

Der Europäische Rat war der zentrale Gestalter des Brexit-Prozesses und der neuen EU-britischen Beziehung. Mit seinen Entscheidungen, die er in ein Narrativ fasste, das die Errungenschaften der EU und die Unterschiede zwischen Mitgliedern und Nichtmitgliedern hervorhob, hat er nicht nur das Verhältnis zum Vereinigten Königreich, sondern wohl auch eine nachhaltig wirkende „Austrittsdoktrin“ (Lippert 2019, S. 632; von Ondarza 2020, S. 92) festgelegt. Somit existiert nun ein Gegenstück zu den Beitrittskriterien, die er 1993 in Kopenhagen formulierte (Europäischer Rat 1993, S. 13). Kernpunkte seiner Festlegungen, die sich zu einer Austrittsdoktrin festigen könnten, sind:

- Ein Nichtmitglied kann nicht bessergestellt sein als ein Mitglied. Wer Rechte haben möchte, muss entsprechende Pflichten übernehmen.
- Teilmitgliedschaften sind nach einem Austritt ebenso wenig möglich (Lippert 2019, S. 632) wie Opt-ins von Nichtmitgliedern in ausgewählte Politikbereiche.
- Der Schutz der Integrität des Binnenmarkts hat höchste Priorität: Eine selektive Beteiligung ist nicht möglich, sondern nur unter Beibehaltung der vier Freiheiten.
- Die Solidarität der Mitglieder untereinander (hier mit Irland) ist ein zentrales Prinzip.
- Das Verfahren und die strategischen Beschlüsse zur Beziehung zum künftigen Ex-Mitglied sind über den unmittelbaren Wortlaut des Vertrags hinaus zur „Chefsache“ (von Ondarza 2020, S. 91) geworden.

Mit seinen Beschlüssen zum Brexit hat der Europäische Rat keine weitere Flexibilisierung der Beteiligungsformen am Integrationsprojekt, die gemäß einer Abbauflexibilisierung denkbar gewesen wäre (Wessels und Wolters 2017, S. 96–97), vorgenommen. Einem „Europe à la carte“ (Dahrendorf 1979), bei dem jeder Staat *Rosinen picken* kann, hat er erneut eine Absage erteilt.

Wie beim vierten Kriterium des Beitrittskatalogs (Europäischer Rat 1993, S. 13) hat er auch hier Entscheidungen getroffen, die die Ausgestaltung der Union selbst betreffen. Er hat das Verfahren in der institutionellen Architektur festgelegt: Durch den Präzedenzfall hat er die Vertragsregeln mit Prägewirkung für die Zukunft interpretiert, mit einer klaren Aufgabenzuteilung an Europäische Kommission, Rat und Europäisches Parlament. Wie in anderen Fällen von zentraler Bedeutung für das EU-System behält er sich das letzte Wort vor, respektiert dabei aber die Rechte von Kommission und Parlament und bindet sie in das Verfahren ein: Nur die Kommission verhandelt mit dem austretenden Staat; das Parlament ist eng beteiligt.

Mit diesen Entscheidungen hat der Europäische Rat auch innerhalb der EU klargestellt, dass die Anwendung von Art. 50 EUV zum Austritt oder Neuverhandlung der Mitgliedschaft, mit dem Ziel, unerwünschte Pflichten loszuwerden, aber Rechte zu behalten, nicht möglich ist (Lippert 2019, S. 634). Inwieweit diese Vorgaben und Festlegungen, zur Doktrin verfestigt, etwaige künftige Austrittskandidaten abschrecken, wird auch davon abhängen, wie sich das EU-britische Verhältnis entwickelt.

Die EU konnte diese Austrittsdoktrin beim Vereinigten Königreich vergleichsweise leicht durchsetzen. Anders als unter Theresa May priorisierte die Johnson-Regierung Souveränität gegenüber wirtschaftlicher Integration und hätte jegliche Art der Teilmitgliedschaft abgelehnt.

Die Verhandlungen zwischen EU und Vereinigtem Königreich sind nicht vorbei. Entscheidungen stehen noch aus und das Abkommen enthält diverse Revisionsklauseln (Bille und Norin 2021). Die künftige Beziehung ist also noch im Fluss. Auf jeden Fall wird jedoch der Europäische Rat bei dessen Gestaltung weiter eine Schlüsselrolle übernehmen wollen.

## Footnotes

1 Im vorliegenden Artikel wird, abweichend vom ZfAS-Standard, bei personenbezogenen Substantiven die männliche grammatikalische Form verwendet. Die Autoren schließen damit Personen jeden Geschlechts gleichermaßen ein.

2 Für die britischen Perspektive auf die Brexit-Verhandlungen und die zukünftigen EU-UK Beziehungen siehe auch den Beitrag von Anand Menon und Matthew Bevington (2021) in dieser Ausgabe.
